# Radical Cystectomy and Ileal Conduit Diversion for Bladder Urothelial Carcinoma With Sarcomatoid and Squamous Variants After Renal Transplantation

**DOI:** 10.7759/cureus.7935

**Published:** 2020-05-02

**Authors:** Abdullah H Yavuzsan, Cumhur Yesildal, Sinan L Kirecci, Musab Ilgi, Ahmet T Albayrak

**Affiliations:** 1 Urology, Sisli Hamidiye Etfal Training and Research Hospital, University of Health Sciences, Istanbul, TUR; 2 Urology, Hopa State Hospital, Artvin, TUR

**Keywords:** kidney transplantation, radical cystectomy, sarcomatoid bladder carcinoma, sarcomatoid carcinoma

## Abstract

Renal transplantation is the optimal treatment for patients with end-stage renal disease. However, the incidence of malignancies, especially urological malignancies, increases after renal transplantation due to long-term immunosuppressive treatments. We report a case of radical cystectomy and ileal conduit diversion in a 39-year-old female patient who developed invasive bladder carcinoma with extravesical extension three years after renal transplantation. Radical cystectomy and ileal conduit diversion surgery are feasible options for patients who developed invasive bladder cancer after renal transplantation and are effective methods for the protection of renal functions in the short-term follow-up period.

## Introduction

Renal transplantation is currently the most appropriate treatment for patients with end-stage renal disease. Graft survival is also prolonged with advanced immunosuppressive therapies [1]. However, prolonged immunosuppression and increased oncogenic virus-induced infections increase the incidence of many malignancies in patients with organ transplantation [2]. In the literature, it has been shown that malignancies are more aggressive and detected at a more advanced stage in organ transplant recipients than the normal population [3,4]. Urological malignancies are among the most common malignancies after renal transplantation [5]. Among urological malignancies, bladder cancer has been shown to be 3.3 times higher in renal transplant recipients [6].

Radical cystectomy and urinary diversion are the standard treatments for muscle-invasive bladder tumors [7]. In this report, we report a radical cystectomy and ileal conduit diversion operation performed in a 39-year-old female patient who was diagnosed three years after renal transplantation due to bladder cancer with extravesical extension.

## Case presentation

**Figure 1:**
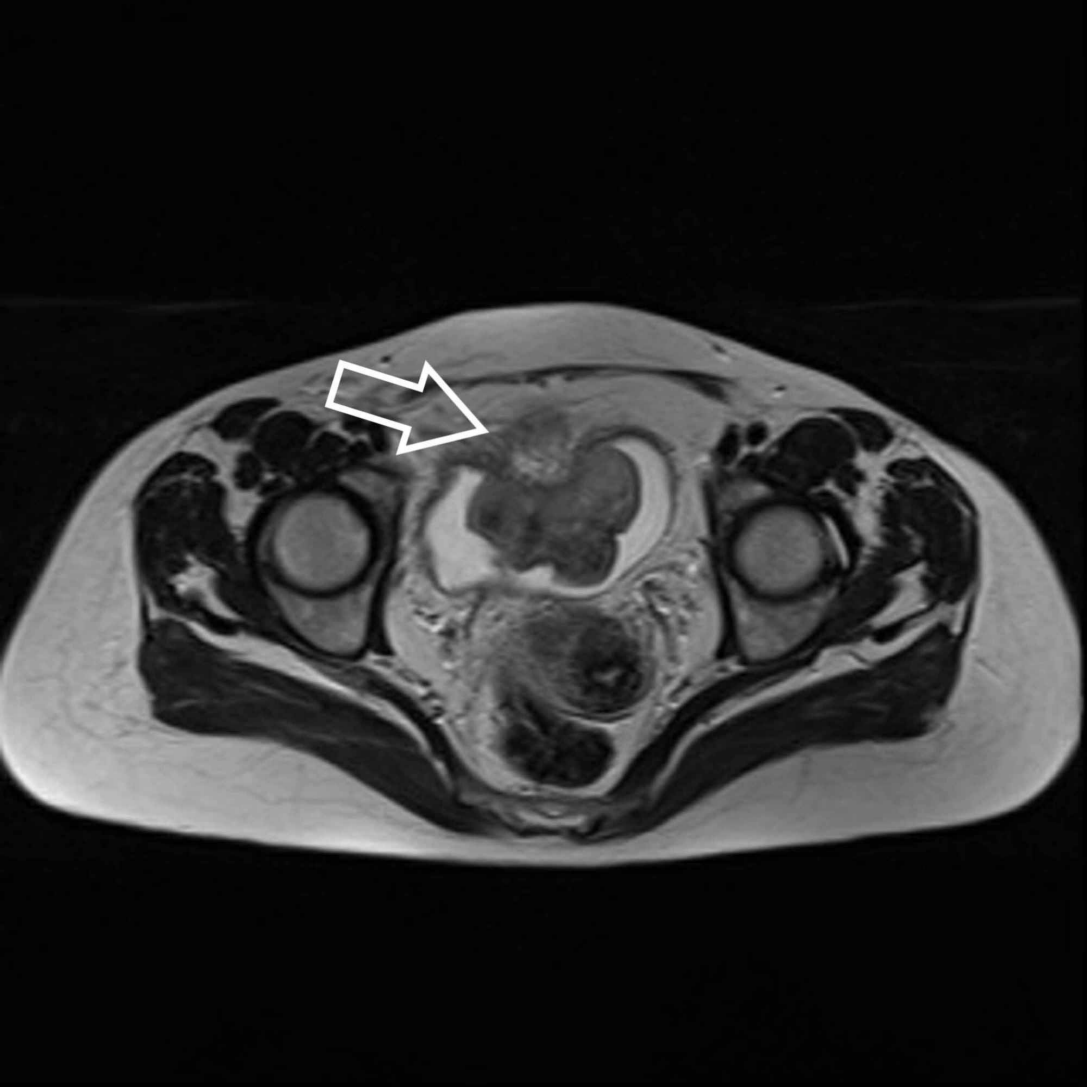
T2-weighted MRI shows protruding mass with extravesical extension (arrow) at the anterior bladder wall. MRI, magnetic resonance imaging

**Figure 2:**
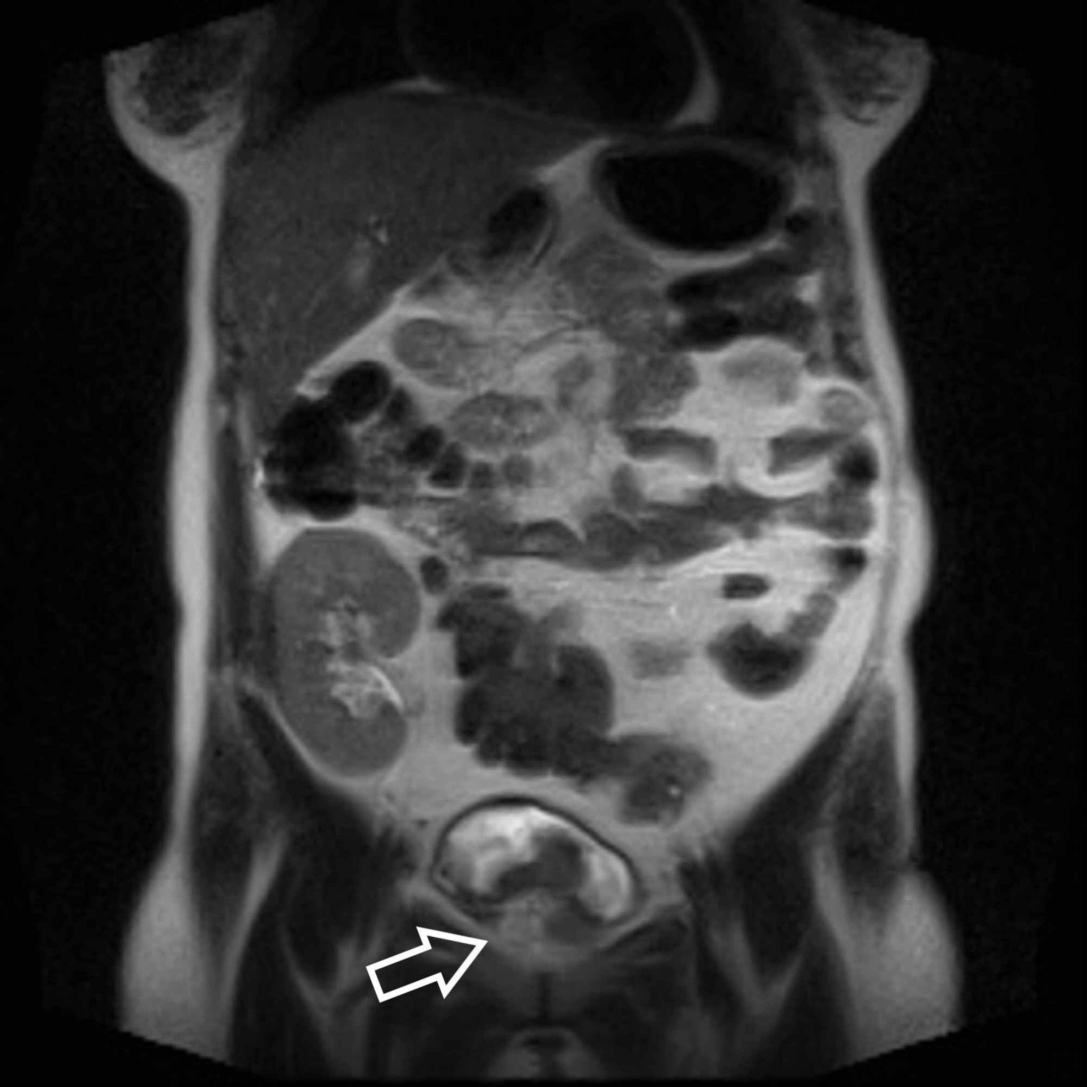
Coronal T2-weighted MRI shows the transplanted kidney in the right iliac fossa and extravesical extension of bladder mass (white arrow). MRI, magnetic resonance imaging

A 39-year-old female patient was admitted to our hospital with macroscopic hematuria. In the history of surgeries, there were right and left nephrectomies due to urolithiasis, and also three years before the admission, there was a living donor renal transplantation. She had a history of hemodialysis for one year before renal transplantation. Serum creatinine level was 1.83 mg/dL (normal: 0.7-1.2 mg/dL). Ultrasonography revealed a 7-cm bladder mass on the anterior wall of the bladder, and the transplanted kidney was normal. Non-contrast abdominal magnetic resonance imaging (MRI) revealed a 36 x 49 x 66 mm mass in the anterior wall of the bladder with extravesical extension, and there was no ectasia or filling defect in the pelvicalyceal structures of the transplanted kidney (Figures 1, 2). Also, there was no metastatic lesion in the solid organs, and non-contrast thoracic computed tomography (CT) scan was normal. A positron emission tomography (PET) CT was not performed because of the previous MRI, and CT revealed no metastatic lesions in relevant areas.

The patient underwent a transurethral resection of the bladder (TURB) to obtain a pathological diagnosis for the tumor. Maximal resection could not be performed due to the tumor size. TURB pathology specimen revealed an infiltrative urothelial carcinoma with sarcomatoid differentiation (pT1). Options of management were discussed with the patient, and a decision was made to proceed with an ileal conduit diversion and radical cystectomy. Renal function of the patient precluded neoadjuvant chemotherapy.

One month after TURB operation, the patient underwent a radical cystectomy and ileal conduit diversion. Pelvic lymph node dissection was performed only on the left side to avoid injury to the transplanted kidney on the right side. The ureter dissection was performed with careful dissection. An approximately 5-cm segment of the distal ureter was dissected while preserving the vascular structures of the transplanted kidney. Uretero-ileal anastomosis was performed in an end-to-end fashion (Wallace technique). The ileal conduit stoma was placed on the right side of the patient and superior to the transplanted kidney. There were no complications in the early postoperative period. Serum creatinine level at discharge was 1.62 mg/dL. Reduction of the dose of immunosuppressive treatment was offered to the patient. However, the patient experienced various dialysis complications and did not accept the risk of graft loss. Eventually, the routine immunosuppressive regimen with methylprednisolone, mycophenolate mofetil, and tacrolimus was continued with the standard dose.

**Figure 3:**
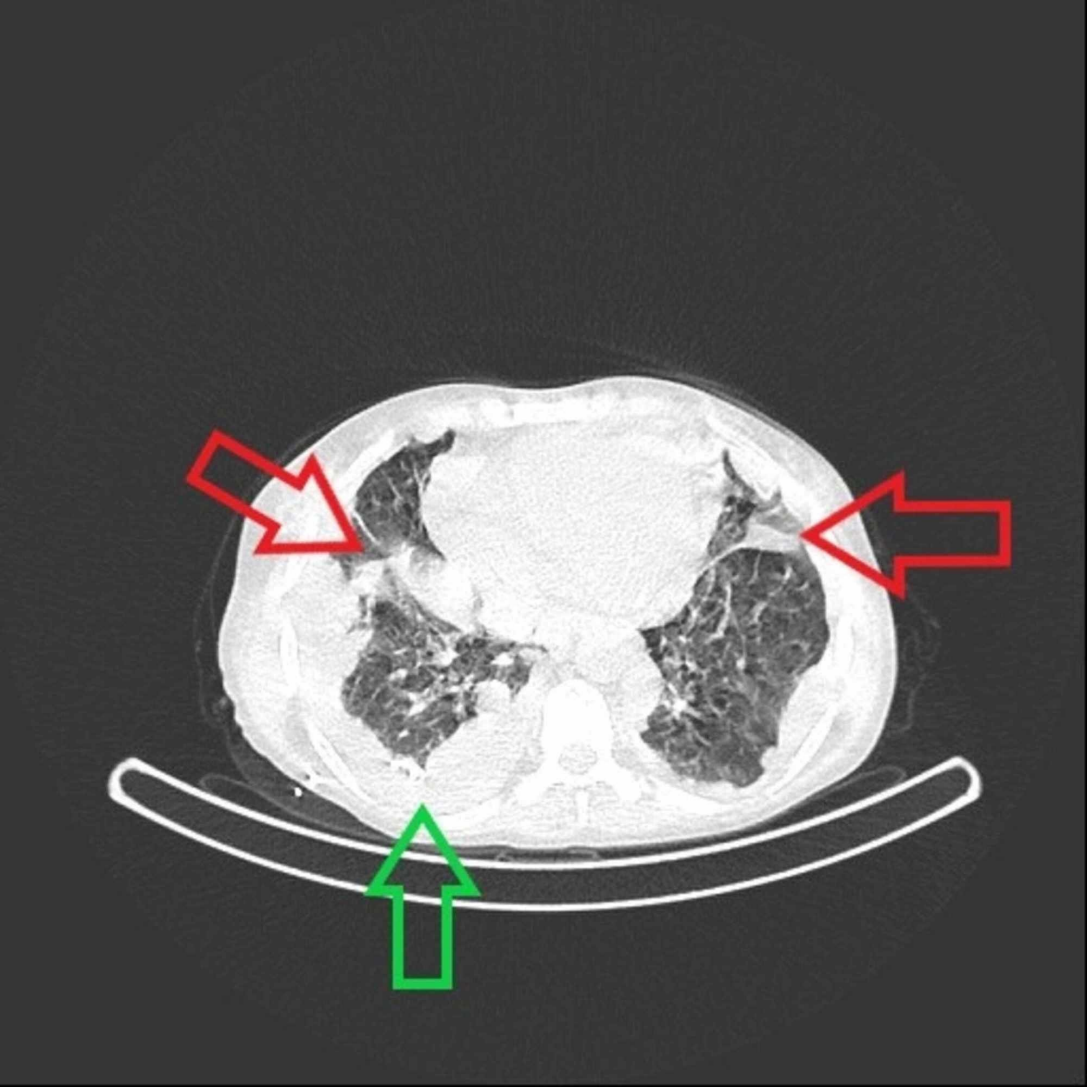
Non-contrast chest CT scan shows bilateral pleural effusion, which is more clear on the right side (green arrow), and bilateral lung consolidations (red arrows) at six months postoperatively. CT, computed tomography

**Figure 4:**
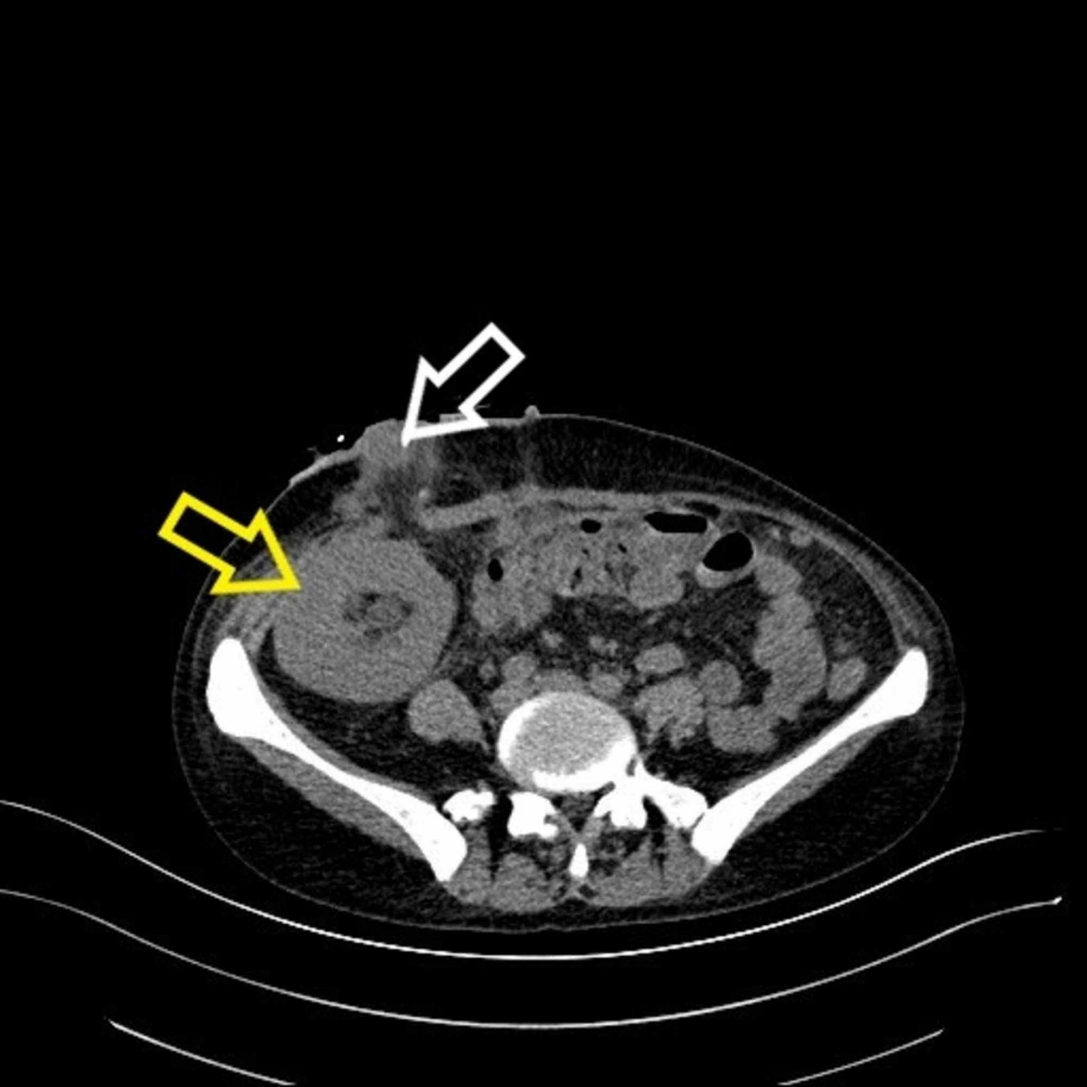
Non-contrast abdominal CT scan shows the transplanted kidney in the right iliac fossa (yellow arrow) and ileal conduit stoma (white arrow). CT, computed tomography

Pathology revealed an infiltrative urothelial carcinoma with sarcomatoid and squamous differentiation (pT3b). The surgical margins were negative, and there was a 1-cm metastatic external iliac lymph node (pN1). Serum creatinine level was 1.58 mg/dL at three months postoperatively, and there was no sign of any metastasis in radiological investigations. The patient presented to the emergency clinic with dyspnea, fever, and fatigue six months after the operation. Her blood creatinine level was 1.53 mg/dL. Bilateral pleural effusion alongside lung consolidations were detected by a thoracoabdominal CT scan (Figure 3). There were no signs of any kind of metastasis, and the transplanted kidney seemed well (Figure 4). The patient was transferred to the intensive care unit due to respiratory failure. An opportunistic Pneumocystis carinii infection was detected in the tracheal aspirate culture. The antibiotic regimen was started by the infectious disease clinic. However, the patient died due to respiratory failure on the 21st day of her admission to the intensive care unit.

This case report has been presented as a poster at the 19th Congress of the European Society for Organ Transplantation in 2019 [8].

## Discussion

Prolonged immunosuppression, chronic antigenic stimulation, viral oncogenesis, and direct carcinogenic effects of immunosuppressive agents result in an increased risk of malignancy in transplant recipients. [9]. Urological malignancies are at the forefront [4,5]. In a comprehensive study, the incidence and prognosis of bladder cancer were investigated in renal transplant recipients and patients with end-stage renal disease [10]. The study showed that the incidence of muscle-invasive bladder cancer (stage T2 or higher) was 37% in renal transplant patients and 24% in patients with end-stage renal disease. Furthermore, it was emphasized that most renal transplant patients were diagnosed as bladder cancer within four years after the transplantation. In our report, the patient was diagnosed with bladder cancer three years after the kidney transplantation.

In de novo malignancies after organ transplantation, it is important to adjust the dose of immunosuppressive therapy. The effects of long-term immunosuppression on the incidence of cancer in renal recipients were compared with a low dose and normal dose of cyclosporine. Although there was no significant difference in graft function and survival, it was reported that there was less malignancy but more organ rejection in the low-dose group [11]. In our case, dose reduction for immunosuppressive therapies was recommended, but the patient did not accept this treatment change due to the risk of graft loss. There was no recurrence of cancer at the six-month postoperative follow-up. Unfortunately, long-term follow-up could not be performed because of the decease of the patient.

The sarcomatoid variant of urothelial carcinoma constitutes less than 1% of all bladder cancers. Sarcomatoid carcinoma is a biphasic malignant tumor with epithelial and mesenchymal differentiation. Due to its aggressive nature, it has a much worse prognosis compared with other variant types. In a study, it was reported that patients with sarcomatoid carcinoma were diagnosed in more advanced stages and had worse disease-specific and overall survival in comparison with urothelial carcinoma [12]. Detection of such an aggressive tumor in organ transplant recipients is more important for the patients. In the literature, studies on the sarcomatoid variant in bladder cancer after kidney transplantation are rare [13,14]. Also, squamous cell bladder carcinoma has been reported in a limited number of cases after kidney transplantation [15,16]. As we know, our case is the first report in the literature to describe both sarcomatoid and squamous variants in bladder cancer after renal transplantation.

If the patient’s medical condition is appropriate for the surgery, the first recommended treatment is radical cystectomy and urinary diversion when the case is detected in muscle invasive or with some high-risk variant histologies of bladder cancer [7]. Urinary diversion can be performed in the form of orthotopic neobladder or an ileal conduit diversion. There is no clear consensus on the type of urinary diversion in patients with renal transplant recipients. However, since orthotopic neobladder is more favorable in terms of patient comfort, orthotopic neobladder has been the preferred method in recent studies [16,17]. However, the orthotopic neobladder method may have more problems such as frequent urinary tract infections due to intermittent catheterization and the development of metabolic complications due to increased urine absorption from the intestinal urinary diversion [17]. In a case presentation, it was reported that a live donor kidney transplantation was performed in a patient who previously had Camey II orthotopic neobladder for genitourinary tuberculosis. It was reported that the patient had urological and metabolic complications since the early posttransplantation period. For this reason, it was suggested that the urinary diversion types to be made should be discussed in detail with the patients, especially for the recipients who have living donors [18]. In our case, urinary diversion methods were discussed thoroughly with the patient who preferred ileal conduit diversion.

## Conclusions

Radical cystectomy and ileal conduit diversion surgery are feasible methods for patients who develop invasive bladder cancer after renal transplantation and are effective methods for the protection of renal functions in the short-term follow-up period. However, because of the ongoing immunosuppressive treatment, patients should be closely monitored, especially for aggressive tumors such as sarcomatoid carcinoma.
